# Repetitive sequences in aging

**DOI:** 10.18632/aging.203020

**Published:** 2021-04-24

**Authors:** Walter Arancio, Claudia Coronnello

**Affiliations:** 1 Advanced Data Analysis Group, Fondazione Ri.MED, Palermo, 90133, Italy

**Keywords:** repetitive sequences, satellites, Alu, LINE-1, HERV, cGAS, STING, Inflammaging, retrotransposons

Repetitive DNA sequences (RS) represent about half of the human genome. Despite since few years ago they have been considered slightly more than “junk DNA”, now RS have a recognized role in almost every aspect of human biology, from embryonic development to infectious diseases.

The role of the RS has been often overlooked because RS are intrinsically difficult to study and specific experimental procedures and bioinformatics pipelines are needed. Each RS is present in thousands or even hundreds of thousands of copies per human genome, and they are often embedded in coding genes and regulatory sequences thus it is extremely difficult to uniquely map them in the reference genome. Moreover, they have accumulated many mutations during evolution and now they diverge into large branched and partially overlapping subfamilies [1].

However, RS can be roughly divided into five classes: i) LTR retroviruses integrated in the genome, the HERV families the most studied among them, that in rare cases can be reactivated; ii) several families of DNA transposons, that are considered evolutionary remnants and are almost inactive; iii) satellite repeats, which are tandem repeats of variable composition and length that are central in the dynamics of centromeres and telomeres but also in the interspersed constitutive heterochromatic regions; iv) the LINEs (long interspersed nuclear elements), which are non-LTR retrotransposons, noteworthy some elements of the LINE-1 family are still retrotransposition-competent and active in humans; v) the SINE (small interspersed nuclear elements), which are non-autonomous retrotransposons that can retrotranspose hijacking the machinery of other retroelements, usually of the LINE-1 family, the most abundant and most studied of them consisting of the primate-specific Alu elements [1].

Interestingly, a plethora of evidences are accumulating in suggesting that RS play a central role in aging and aging related diseases. In particular, it is a consolidated knowledge that the RS at the telomeres are strictly interlaced with the replicative senescence and the satellites in general with chromosomal stability, especially those present in the centromeric regions.

But even more, it is known that RS are under a strict epigenetic control mechanism based on CpG DNA methylation. Indeed, most of the genomic methylated CpG sites are carried by RS. During aging, this epigenetic landscape is subverted, and the sequence homologies of RS became a hub for homology directed recombination thus causing in turn genomic instability, DNA damage, senescence and even cancer transformation. Moreover, RS usually carry regulatory sequences, thus their deregulation, or mobilization, can alter the transcriptional profile of the cell [2].

Alteration of the cellular homeostasis caused by RS can be mediated not only by their epigenetic state but also by their transcriptional activity. Even if RS were considered almost transcriptional silent, it is now evident that they are actively transcribed, and that their transcription is strictly regulated and have a large impact in the biology of the cell.

A recent paper [3] quantified the global RS transcription profile in a model of aging of dopaminergic neurons where the expression of progerin was induced. Progerin is the protein whose accumulation in the nuclear membrane causes the Hutchinson–Gilford progeria syndrome, but it also accumulates during the canonical aging process. Unexpectedly, a global downregulation of the vast majority of RS transcripts was reported in the model. Interestingly, this global downregulation is specific to neurons, and fibroblasts showed a different behavior, where instead the expression of specific RS is altered. This is not surprising, because the role of RS in brain is quite peculiar; indeed, retrotransposition of LINE-1 element is physiologically significantly high in neuronal precursor cells [4] leading to genomic brain mosaicism in human adults. The specific role of RS mobilization in the developing brain is not fully understood, although it seems necessary to the structural and functional organization of the adult brain. However, uncontrolled RS activities have been proposed as potential players in neurodegenerative diseases, such as Alzheimer’s disease [5]. In particular, the dysregulation of Alu elements alters mitochondrial homeostasis and their increased expression contributes to inflammation within the nervous system [6].

More generally, RS activation has been suggested as a general feature of aging cells that acquire the senescent phenotype [7] and recently [8] it has been proved that LINE-1 derepression in senescent cells triggers the type-I interferon (IFN-I) pathway and the inflammation associated with aging (inflammaging). In particular, the authors showed that during the process of replicative senescence, and with minor differences also in oncogene-induced and stress-induced senescence, LINE-1 elements are derepressed and are activated and transcribed exponentially during the process. The accumulation of cDNA derived from LINE-1 elements in the cytosol triggers the cytosolic DNA sensing pathway, a component of the innate immune system against virus infection. Upon LINE-1 cDNA binding, cGAS becomes enzymatically active and generate the second messenger cGAMP that in turn activates STING (Stimulator of interferon genes), and thus triggers the IFN-I response. The IFN-I response has been proposed as a key player in the maintenance of the SASP (senescence associated secretory phenotype) during the late senescence, that lies at the core of inflammaging. The authors even suggest that RS are relevant potential targets for the treatment of age-associated disorders.

Overall, it is becoming clearer day by day that the study of RS dynamics is crucial in the understanding of cellular homeostasis and its deregulation, especially in the contest of genomic instability and cellular senescence that underlie the process of human aging.
